# α_7_ Nicotinic Agonist AR-R17779 Protects Mice against 2,4,6-Trinitrobenzene Sulfonic Acid-Induced Colitis in a Spleen-Dependent Way

**DOI:** 10.3389/fphar.2017.00809

**Published:** 2017-11-08

**Authors:** Andrea Grandi, Irene Zini, Lisa Flammini, Anna M. Cantoni, Valentina Vivo, Vigilio Ballabeni, Elisabetta Barocelli, Simona Bertoni

**Affiliations:** ^1^Food and Drug Department, University of Parma, Parma, Italy; ^2^Department of Veterinary Sciences, University of Parma, Parma, Italy

**Keywords:** intestine, inflammation, α_7_ nicotinic agonist, α_4_β_2_ nicotinic receptors, sulfasalazine, lymph nodes

## Abstract

The existence of a cholinergic anti-inflammatory pathway negatively modulating the inflammatory and immune responses in various clinical conditions and experimental models has long been postulated. In particular, the protective involvement of the vagus nerve and of nicotinic Ach receptors (nAChRs) has been proposed in intestinal inflammation and repeatedly investigated in DSS- and TNBS-induced colitis. However, the role of α_7_ nAChRs stimulation is still controversial and the potential contribution of α_4_β_2_ nAChRs has never been explored in this experimental condition. Our aims were therefore to pharmacologically investigate the role played by both α_7_ and α_4_β_2_ nAChRs in the modulation of the local and systemic inflammatory responses activated in TNBS-induced colitis in mice and to assess the involvement of the spleen in nicotinic responses. To this end, TNBS-exposed mice were sub-acutely treated with various subcutaneous doses of highly selective agonists (AR-R17779 and TC-2403) and antagonists (methyllycaconitine and dihydro-β-erythroidine) of α_7_ and α_4_β_2_ nAChRs, respectively, or with sulfasalazine 50 mg/kg per os and clinical and inflammatory responses were evaluated by means of biochemical, histological and flow cytometry assays. α_4_β_2_ ligands evoked weak and contradictory effects, while α_7_ nAChR agonist AR-R17779 emerged as the most beneficial treatment, able to attenuate several local markers of colitis severity and to revert the rise in splenic T-cells and in colonic inflammatory cytokines levels induced by haptenization. After splenectomy, AR-R17779 lost its protective effects, demonstrating for the first time that, in TNBS-model of experimental colitis, the anti-inflammatory effect of exogenous α_7_ nAChR stimulation is strictly spleen-dependent. Our findings showed that the selective α_7_ nAChRs agonist AR-R17779 exerted beneficial effects in a model of intestinal inflammation characterized by activation of the adaptive immune system and that the spleen is essential to mediate this cholinergic protection.

## Introduction

During the last two decades, the existence of a CAP, vagus- and spleen-dependent, has been postulated by several authors (Olofsson et al., 2012; Bonaz et al., 2013; Martelli et al., 2014; Goverse et al., 2016). Apparently pivotal to the anti-inflammatory actions of CAP is the stimulation of α_7_ nAChR homomeric subtype (Wang et al., 2003). α_7_ nAChRs are expressed in several regions of the central nervous system and, in the periphery, on neurons, like the adrenergic neurons in the celiac/superior mesenteric ganglia and splenic nerve fibers (Downs et al., 2014), and non-neuronal cells like gut epithelial and glial cells (Costantini et al., 2012), endothelial cells (Saeed et al., 2005) and immune cells, such as macrophages (Wang et al., 2003), T lymphocytes, and dendritic cells (Kawashima et al., 2012). In particular, their activation by nicotine on immune cells can incite an intracellular cascade via Jak2/STAT3 signaling pathway leading to the inhibition of NF-κB transcription factor and, consequently, to the negative regulation of pro-inflammatory cytokines secretion (De Jonge et al., 2005). Accordingly, the anti-inflammatory properties of various selective α_7_ nAChR agonists have been demonstrated in a number of models of acute inflammation *in vivo*, such as acute lung injury (Pinheiro et al., 2017), cardiopulmonary bypass (Ge et al., 2016), burn injury (Kashiwagi et al., 2017), mesenteric and renal ischemia/reperfusion (Li et al., 2013; He et al., 2016), sepsis (Nullens et al., 2016) and post-operative ileus (The et al., 2007). More controversial are the effects produced by α_7_ nAChR agonists on subacute/chronic intestinal inflammation: in dextran sulfate sodium (DSS)-induced colitis, a model mainly dependent on the activation of innate immune responses, alternatively a protective (Salaga et al., 2016), anti-hyperalgesic, but not anti-inflammatory (Costa et al., 2012), or even detrimental (Snoek et al., 2010) role has been evidenced for α_7_ nAChRs stimulation, while in TNBS-induced colitis, where adaptive immune responses prevail (Kiesler et al., 2015), α_7_ nAChR agonists seem to exert a protective activity only when acting as partial agonists (Snoek et al., 2010; Salaga et al., 2016).

More recently, a potential counter-inflammatory action has emerged also for α_4_β_2_ nAChRs. Heteromeric α_4_β_2_ nAChRs, the predominant high-affinity binding sites for nicotine in the central nervous system, are well known to mediate the reinforcing, rewarding and dependence-inducing properties of nicotine (De Biasi and Dani, 2011) and its anti-nociceptive actions (Hurst et al., 2013). In the periphery, α_4_β_2_ nAChRs have been identified also on non-neuronal cells, like peritoneal, intestinal (van der Zanden et al., 2009) and alveolar macrophages (Matsunaga et al., 2001): their stimulation may inhibit the release of pro-inflammatory cytokines production through NF-κB inhibition in transfected human cells (Hosur and Loring, 2011) and promote the phagocytic activity of isolated intestinal and peritoneal murine macrophages (van der Zanden et al., 2009). However, no information is available up to now about the ability of α_4_β_2_ nAChRs ligands to modulate intestinal inflammation *in vivo*, as in colitis, where macrophages purportedly play a pivotal role (Mahida et al., 1989).

Starting from these premises, the main aim of the present study was to pharmacologically investigate the role of α_7_ and α_4_β_2_ nAChRs subtypes in the modulation of the local and systemic inflammatory responses in a murine model of colitis characterized by the pronounced activation of the adaptive immune system. To this purpose, we evaluated the effects produced by highly selective agonists (AR-R17779 and TC-2403) (Bencherif et al., 1996; Mullen et al., 2000) and antagonists (MLA and DBE) (Ward et al., 1990; Niimi et al., 2013) of α_7_ and α_4_β_2_ nAChRs, respectively, on the clinical and inflammatory markers increased in mice by TNBS colitis. The α_7_ nAChR agonist AR-R17779 emerged as the most effective treatment, able to attenuate colitis severity and to revert the rise in splenic T-cells populations and in colonic inflammatory cytokines levels induced by haptenization.

In view of the prominent role played by both the spleen and α_7_ nAChR signaling in mediating the CAP, we asked whether the protection afforded by AR-R17779 against TNBS-induced colitis could be maintained also after SPX and we demonstrated that, in our model of experimental colitis, the presence of the spleen is essential to the anti-inflammatory effect of exogenous α_7_ nAChR stimulation.

## Materials and Methods

### Animals

All animal experiments were performed according to the guidelines for the use and care of laboratory animals and they were authorized by the local Animal Care Committee “Organismo Preposto al Benessere degli Animali” and by Italian Ministry of Health, “Ministero della Salute” (DL 26/2014). Animal studies are reported in compliance with the ARRIVE guidelines (Kilkenny et al., 2010; Curtis et al., 2015). All appropriate measures were taken to minimize pain or discomfort of animals. Female CD1 Swiss mice (7–12 weeks old) (Charles River Laboratories, Calco, Italy), weighing 25–30 g, were housed five per cage and maintained under standard conditions at our animal facility (12:12 h light–dark cycle, water and food *ad libitum*, 22–24°C). Intragastric gavage administration was performed using suitable gavage needles (22 gauge, length 3 cm, ball diameter 1.5 mm). All the experimental procedures (induction of colitis, SPX) and euthanasia by CO_2_ inhalation were performed between 9 a.m. and 12 a.m.

### Induction of Colitis

Six days before intrarectal (i.r.) instillation, animals were subjected to skin sensitization through cutaneous application of 50 μL of a 10% (w v^-1^) TNBS solution in 50% ethanol. After 20 h fasting with free access to water containing 5% glucose, colitis was induced by i.r. instillation of the same volume and concentration of TNBS applied during skin sensitization (Waldner and Neurath, 2009). TNBS instillation was performed using a PE50 catheter, positioned 4 cm from the anus, in anesthetized mice (isoflurane 2%) kept in the head-down position for 3 min to avoid the leakage of intracolonic instillate. The TNBS-induced colitis model with previous skin sensitization was chosen to have a model of colitis characterized by the prominent activation of the adaptive immune system (Te Velde et al., 2006).

### Splenectomy

In a second series of experiments, chemical colitis was induced in mice 15 days after SPX. Mice, fasted for 16 h, were anesthetized by intraperitoneal injection of pentobarbital 60 mg⋅kg^-1^. The spleen was removed after laparotomy and ligation of blood vessels and, after the surgical intervention, mice were monitored daily to examine their health state and scar cicatrisation.

### Experimental Design

Pharmacological treatments started 8 h after TNBS enema (day 1) and were applied once (sulfasalazine) or twice daily (b.i.d.), 8 h apart, during the following 2 days by a researcher not involved in biochemical, histological assays or daily monitoring of Disease Activity Index. All the pharmacological treatments were subcutaneously (s.c.) administered except for sulfasalazine, per os (p.o.) applied. Animals were euthanized by CO_2_ inhalation 3 days after TNBS or saline instillation (day 4).

Mice were assigned through block randomization to the normal group (N; *n* = 24), i.r. inoculated with 50 μL 0.9% NaCl (saline solution) and administered s.c. 10 mL kg^-1^ saline (b.i.d.) or to the following experimental groups of colitic mice: saline (CTR, 10 mL kg^-1^; *n* = 28), sulfasalazine 50 mg kg^-1^ die^-1^ p.o. (S; *n* = 7), TC-2403 2 mg kg^-1^ (*n* = 7) and 5 mg kg^-1^ (*n* = 7), DBE 0.5 mg kg^-1^ (*n* = 7) and 1.5 mg kg^-1^ (*n* = 7), AR-R17779 (A) 0.5 mg kg^-1^ (*n* = 7), 1.5 mg kg^-1^ (*n* = 16), 5 mg kg^-1^ (*n* = 7), MLA 0.1 mg kg^-1^ (*n* = 7), 0.5 mg kg^-1^ (*n* = 7), 1 mg kg^-1^ (*n* = 7). The dosage of each pharmacological treatment was chosen on the basis of the literature (Karimi et al., 2010; Snoek et al., 2010; Joh and Kim, 2011; Hijioka et al., 2012; Niimi et al., 2013). The study was performed using experimental blocks composed by 10 or 12 mice that were randomly assigned to five or six groups of treatment (N and CTR were present in each experimental block), each one encompassing two animals.

Splenectomy mice were assigned through block randomization to the following experimental groups: SPX/N (saline i.r., 10 mL kg^-1^ saline; *n* = 15), SPX/C (TNBS i.r., 10 mL kg^-1^ saline; *n* = 15) and SPX/A (TNBS i.r., AR-R17779 1.5 mg kg^-1^; *n* = 15). SPX study was performed through three experimental blocks composed by 15 mice that were randomly assigned to three groups of treatment, each one comprising five animals.

A group size of *n* = 7 was determined according to “*a priori* power analysis,” by setting α = 0.05, 1-β = 0.8 and effect size = 0.6 assuming as primary endpoint the reduction of colonic MPO levels. Due to seasonal variability, N and CTR mice were repeated periodically all over the study in order to verify the attainment of a constant degree of colitis severity with respect to physiological conditions, thus explaining the bigger size of N and CTR experimental groups with respect to the other groups. As regards A1.5, SPX/N, SPX/C, and SPX/A groups, a higher number of mice was necessary to perform the histological and biochemical assays described. Accordingly, each group of animals was randomly subdivided in two subgroups: colons excised from each subset was reserved either for histological analysis or for MPO activity determination and for cytokines assays. In this latter case, the colon was sometimes longitudinally split in two halves, each one dedicated to MPO activity assay or to cytokines determination.

### Evaluation of Inflammatory Responses

Body weight, stools consistency and rectal bleeding were examined and registered daily throughout the experimentation by unaware observers, in order to assess the DAI. Immediately after euthanasia the macroscopic damage of colonic mucosa was assessed as MS. The wet weight and the length of each colon were measured and the weight/length ratio was considered as disease-related intestinal wall thickening (Bischoff et al., 2009). Colon, lungs, spleen and MLNs were collected for subsequent microscopic, biochemical or flow cytometry analyses.

#### Disease Activity Index

Disease Activity Index is a parameter that estimates the severity of the disease; it is based on the daily assignment of a total score, according to Cooper’s modified method (Cooper et al., 1993), on the basis of body weight loss, stool consistency and rectal bleeding. The scores were attributed blindly by two investigators and were quantified as follows: stool consistency: 0 (normal), 1 (soft), 2 (liquid); body weight loss: 0 (<5%), 1 (5–10%), 2 (10–15%), 3 (15–20%), 4 (20–25%), 5 (>25%) and rectal bleeding: 0 (absent), 1 (present).

#### Colon Macroscopic Damage Score (MS)

After euthanasia, the colon was explanted, opened longitudinally, flushed with saline solution and MS was immediately evaluated through inspection of the mucosa, executed by two investigators unaware of the treatments applied. MS was determined according to previously published criteria (Rapalli et al., 2016), as the sum of scores (max = 12) attributed as follows: presence of strictures and hypertrophic zones (0, absent; 1, one stricture; 2, two strictures; 3, more than two strictures); mucus (0, absent; 1, present); adhesion areas between the colon and other intra-abdominal organs (0, absent; 1, one adhesion area; 2, two adhesion areas; 3, more than two adhesion areas); intraluminal hemorrhage (0, absent; 1, present); erythema (0, absent; 1, presence of a crimsoned area < 1 cm^2^; 2, presence of a crimsoned area > 1 cm^2^); ulcerations and necrotic areas (0, absent; 1, presence of a necrotic area < 1 cm^2^; 2, presence of a necrotic area > 1 cm^2^).

#### Colonic Length and Thickness

To evaluate muscular contraction and deposition of fibrotic material induced by a prolonged inflammatory state, the length of the colon and its weight were measured, while weight/length ratio was calculated to estimate colon thickness, according to a previously published method (Bischoff et al., 2009).

#### Colonic and Pulmonary Myeloperoxidase Activity Assay

Myeloperoxidase activity, marker of granulocytic infiltration within a tissue, was determined according to Krawisz’s modified method (Krawisz et al., 1984). After being weighed, each colonic or lung sample was homogenized in ice-cold potassium phosphate buffer (100 mmolL^-1^, pH 7.4) containing 1 μg mL^-1^ aprotinin and centrifuged for 20 min at 12500 RCF at 4°C. Pellets were re-homogenized in five volumes of ice-cold potassium phosphate buffer (50 mmolL^-1^, pH 6) containing 0.5% hexadecyltrimethylammonium bromide (HTAB) and 1 μg mL^-1^ aprotinin. Samples were then subjected to three cycles of freezing and thawing and centrifuged for 30 min at 15500 RCF at 4°C. One hundred microliters of the supernatant was then allowed to react with 900 μL of a buffer solution containing 0.167 mg mL^-1^ *o*-dianisidine and 0.0005% H_2_O_2_. Each assay was performed in duplicate and the rate of change in absorbance was measured spectrophotometrically at 470 nm (Jenway, mod. 6300, Dunmow, Essex, England). The sensitivity of the assay was 10 mU mL^-1^, 1 unit of MPO being defined as the quantity of enzyme degrading 1 μmol of peroxide per minute at 25°C. Data were normalized with edema values [(wet weight – dry weight) dry weight^-1^] (Moore-Olufemi et al., 2005) and expressed as U g^-1^ of dry weight tissue.

### Flow Cytometry Assays

#### Isolation of Splenocytes

Spleen, explanted immediately after euthanasia, was mechanically dispersed through a 100 μm cell-strainer and washed with PBS containing 0.6 mmolL^-1^ EDTA (PBS-EDTA). The cellular suspension was then centrifuged at 1500 RCF for 10 min at 4°C, the pellet re-suspended in PBS-EDTA and incubated with 2 mL of NH_4_Cl lysis buffer (0.15 molL^-1^ NH_4_Cl, 1 mmolL^-1^ KHCO_3_, 0.1 mmolL^-1^ EDTA in distilled water) for 5 min, in the dark, to induce the lysis of erythrocytes. Afterward, samples were centrifuged at 1500 RCF for 10 min at 4°C, the pellet was washed with PBS-EDTA and re-suspended in 5 mL cell staining buffer (PBS containing 0.5% FCS and 0.1% sodium azide). The obtained cellular suspension was stained with fluorescent antibodies (Kruisbeek, 2001).

#### Isolation of Mesenteric Lymph Nodes

The lymphoid tissue located in the middle of proximal colon’s mesentery was explanted immediately after euthanasia and flushed with PBS, then MLNs were isolated from adherent adipose and vascular tissue, mechanically dispersed through a 100 μm cell-strainer and washed with Hank’s Balanced Salt Solution (HBSS) containing 5% FCS. The cellular suspension was centrifuged at 1500 RCF for 10 min at 4°C, the pellet washed with HBSS containing 5% FCS and re-suspended in 3 mL cell staining buffer. The obtained cellular suspension was subjected to staining with fluorescent antibodies (Lopez, 2007).

#### Immunofluorescent Staining

Before the incubation with fluorescent antibodies, the cellular suspension of splenocytes or MLN was incubated with IgG1-Fc (1 μg 10^-6^ cells) for 10 min in the dark at 4°C, in order to block non-specific binding sites for antibodies. The following antibodies were used: Phycoerythrin-Cyanine 5 (PE-Cy5) conjugated anti-mouse CD3ε (0.25 μg 10^-6^ cells), Fluorescein Isothiocyanate (FITC) anti-mouse CD4 (0.25 μg 10^-6^ cells), PE anti-mouse CD8a (0.25 μg 10^-6^ cells), FITC anti-mouse F4/80 (0.25 μg 10^-6^ cells), Peridinin Chlorophyll Proteins-Cyanine5.5 (PerCP-Cy5.5) anti-mouse CD25 (1 μg 10^-6^ cells) and PE anti-mouse FoxP3 (1 μg 10^-6^ cells). Cells were incubated with antibodies for 1 h in the dark at 4°C, washed with PBS to remove excessive antibody and suspended in cell staining buffer to perform flow cytometry analysis. Because of the intracellular localization of FoxP3, staining with PE anti-mouse FoxP3 was preceded by cells fixation and permeabilization: after staining with cell surface markers, cells were fixed with FOXP3 Fix/Perm Buffer and permeabilized with PBS containing 0.2% Tween 20. Cells were then incubated with PE anti-mouse FoxP3 for 30 min in the dark, at room temperature, washed with PBS to remove excessive antibody and suspended in cell staining buffer (Holmes et al., 2001).

The viability of the cellular suspension was assessed through propidium iodide (PI) staining, a membrane impermeable fluorescent dye, excluded by viable cells, that binds to DNA emitting red fluorescence, thus resulting as a suitable marker for dead cells. Cells were incubated with 10 μg mL^-1^ PI for 1 min in the dark, at room temperature, and immediately subjected to flow cytometry analysis. Only PI^-^ cells were included in the analysis.

Samples were analyzed using Guava easyCyte^TM^ and InCyte^TM^ software (Merck Millipore, Darmstadt, Germany). The various spleen and MLN cell populations were defined as follows: lymphocytes gated in the Forward Scatter (FSC)-Side Scatter (SSC) plot as FSC low: SSC low; T lymphocytes (CD3^+^); CD4^+^ T lymphocytes (CD3^+^CD4^+^CD8^-^); CD8^+^ T lymphocytes (CD3^+^CD8^+^CD4^-^); CD4^+^ Tregs (CD4^+^CD25^+^FoxP3^+^); macrophages (CD3^-^F4/80^+^). Percentages were reported to the total number of splenocytes or MLN cells of each mouse to calculate the number of cells per population.

### Colonic Cytokines Levels

After euthanasia, colon tissues were homogenized for 1 min in tissue lysis buffer containing protease inhibitor cocktail (aprotinin 1 μg mL^-1^ and leupeptin 1 μg mL^-1^). Samples were then centrifuged for 30 min at 14000 RCF and the supernatant was collected. Total protein concentration was quantified using Pierce BCA protein assay kit (Thermo Fisher Scientific Inc., Waltham, MA, United States). IL-1β, IL-6, IFNγ, and IL-10 levels were then determined in duplicate in 100 μL samples, using commercially available ELISA kits (Mouse IL-10 ELISA kit, Abcam Biochemicals^TM^, Cambridge, United Kingdom; Mouse IL-1β ELISA kit; Mouse IFNγ ELISA kit; Mouse IL-6 ELISA kit, RayBio^TM^, Norcross, GA, United States) according to the manufacturer’s protocol. The absorbances of the samples were measured spectrophotometrically at 450 nm (TECAN Sunrise^TM^ powered by Magellan^TM^ data analysis software, Mannedorf, Switzerland). The assays sensitivity were, respectively, 5 pg mL^-1^ (IL-1β), 2 pg mL^-1^ (IL-6), 10 pg mL^-1^ (IFNγ), and 30 pg mL^-1^ (IL-10). Results were expressed as pg mg^-1^ protein.

### Colonic Histology

Colonic samples were harvested from N, CTR, A1.5, SPX/N animals and from colitic splenectomized mice, either administered with saline (SPX/C) or with AR-R17779 1.5 mg/kg (SPX/A), immersion-fixed in 10% neutral buffered formalin overnight, dehydrated and embedded in paraffin. For each sample, at least five transverse 5-μm sections were cut in the distal colon, stained with hematoxylin–eosin and blindly examined in a light microscope (Nikon Eclipse E800). The histological damage was quantified according to Rapalli et al. (2016) by an unaware investigator: the grade of mucosal destruction (0, normal; 1, mild; 2, moderate; 3, severe) and the degree of leukocytes infiltration in the lamina propria and submucosa (0, absent; 1, mild; 2, pronounced) were scored (maximum score: 7). Since histological injury produced by TNBS instillation was localized almost exclusively in the distal portion of the colon, the average value of histological score was determined only from sections of distal colon, pooled with those determined for the other animals of the same experimental group and the mean value ± SEM was calculated.

### Statistical Analysis

The data and statistical analysis comply with the recommendations on experimental design and analysis in pharmacology (Curtis et al., 2015). All data, each one representing the outcome of a single animal, were presented as means ± SEM. Comparisons among experimental groups were made using analysis of variance (one-way or two-way ANOVA) followed by Bonferroni’s post-test when *P* < 0.05, chosen as level of statistical significance, was achieved. All analyses were performed using Prism 4 software (GraphPad Software Inc., San Diego, CA, United States).

### Materials

Sulfasalazine, MLA, TNBS, ethanol, HTAB and 30% hydrogen peroxide were purchased from Sigma–Aldrich^TM^ (St. Louis, MO, United States). AR-R17779 and TC-2403 were purchased from Abcam Biochemicals^TM^ (Cambridge, United Kingdom), while DBE was purchased from Tocris Bioscience^TM^ (Bristol, United Kingdom). Drugs were dissolved in saline solution the day of the experiment. Fluorescent antibodies used for flow cytometry (FITC anti-mouse CD4, PE anti-mouse CD8, PE anti-mouse FOXP3, PerCP/Cy5.5 anti-mouse CD25), PI and FOXP3 Fix/Perm Buffer were purchased from BioLegend^TM^ (San Diego, CA, United States), PE-Cy5 anti-mouse CD3 from Affymetrix eBioscience^TM^ (San Diego, CA, United States) and IgG1-Fc from Millipore^TM^ (Merck, Darmstadt, Germany).

## Results

### TNBS Triggered a Severe Inflammatory Response Controlled by Sulfasalazine

**FIGURE 1:**
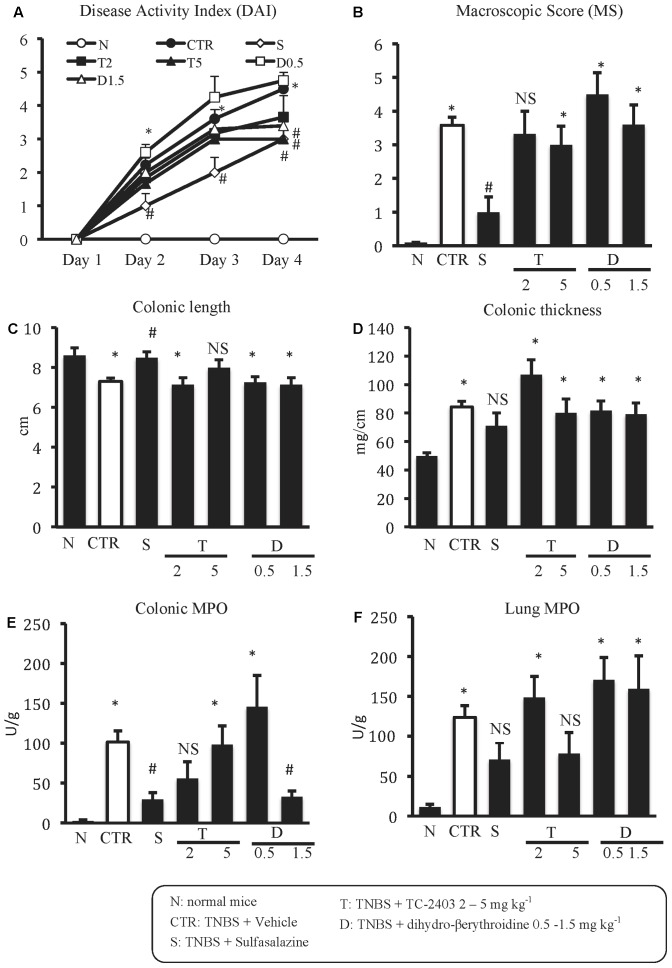
Effects of sulfasalazine and α_4_β_2_ nAChRs ligands on TNBS-induced inflammatory responses. Disease Activity Index **(A)**, macroscopic score **(B)**, colonic length **(C)**, colonic thickness **(D)**, colonic MPO **(E)** and lung MPO **(F)** activity assessed in vehicle-treated normal mice (N) and in TNBS-treated mice administered with vehicle (CTR – white bar), sulfasalazine 50 mg/kg (S), TC-2403 (T) 2 and 5 mg/kg, DBE (D) 0.5 and 1.5 mg/kg (D1.5) (*n* = 7–12 independent values per group). ^∗^*P* < 0.05 vs. N mice; ^#^*P* < 0.05 vs. CTR mice; NS, not significant vs. N and CTR mice; one-way ANOVA followed by Bonferroni’s post-test.

**FIGURE 2:**
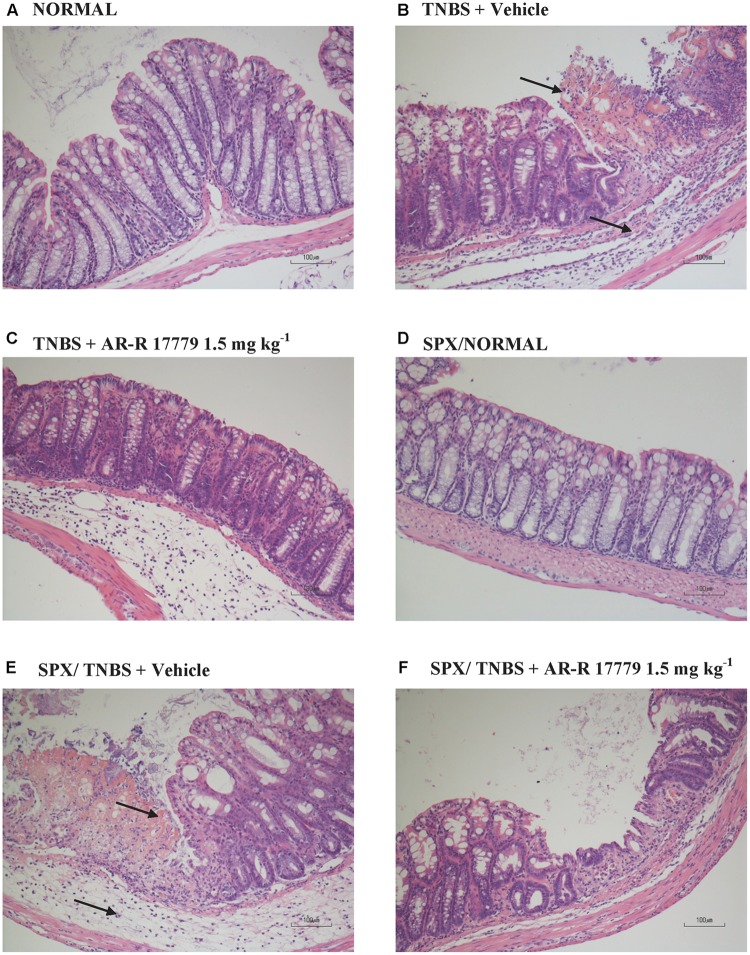
Histology. Representative hematoxylin–eosin stained sections of colonic specimens harvested from vehicle-treated normal mice, non-operated **(A)** or splenectomized **(D)**, and from TNBS-treated mice administered with vehicle, non-operated **(B)** or splenectomized **(E)**, or with AR-R17779 1.5 mg/kg, non-operated **(C)** or splenectomized **(F)**. TNBS colonic instillation caused mucosal necrosis, neutrophils infiltration and submucosal oedema (indicated by arrows) in vehicle-treated animals **(B,E)**, attenuated by AR-R17779 treatment in non-operated mice **(C)** but not in splenectomized animals **(F)**.

Animals subjected to hapten-induced colitis showed a marked decrease of body weight and reduction of stools’ consistency compared with normal mice, as indicated by DAI increase from day 1 to day 4 (**Figure 1A**). Locally, TNBS instillation provoked a strong shortening and thickening of the colon and a substantial increase of macroscopic damage score, due to the appearance of ulcerations, luminal hemorrhages and erythemas (**Figures 1B–D**). Accordingly, histological observation revealed extensive mucosal necrosis and abundant granulocytes infiltration both in the lamina propria and in the submucosa in TNBS-exposed mice (**Figure 2B**; histological score: 6.5 ± 0.3 in Supplementary Figure 1A) compared with normal mice (**Figure 2A**; histological score: 1.0 ± 0.6 in Supplementary Figure 1A). Finally, upon instillation of the haptenating agent we observed a consistent granulocyte infiltration both in the colon and within the lungs (**Figures 1E,F**).

As expected, treatment with the anti-inflammatory agent sulfasalazine was able to dampen the local and systemic inflammatory responses induced by TNBS, improving mice health conditions by counteracting both the weight loss and diarrhea over the whole treatment period (**Figure 1A**), decreasing colon shortening and mucosal damage and mitigating colonic neutrophil infiltration (**Figures 1B,C,E**).

### α_4_β_2_ nAChRs Agents Evoked Weak Effects on Experimental Colitis

The repeated administration of α_4_β_2_ nAChRs agents in colitic mice elicited weak effects on most of the analyzed inflammatory parameters. Only DAI was significantly reduced on day 4 by both the agonist TC-2403 and the antagonist DBE, when administered at the highest tested dose (**Figure 1A**); at the same dose, DBE was able to attenuate also colonic MPO activity (**Figure 1E**).

### α_7_ nAChRs Agonist Protects against Experimental Colitis

**FIGURE 3:**
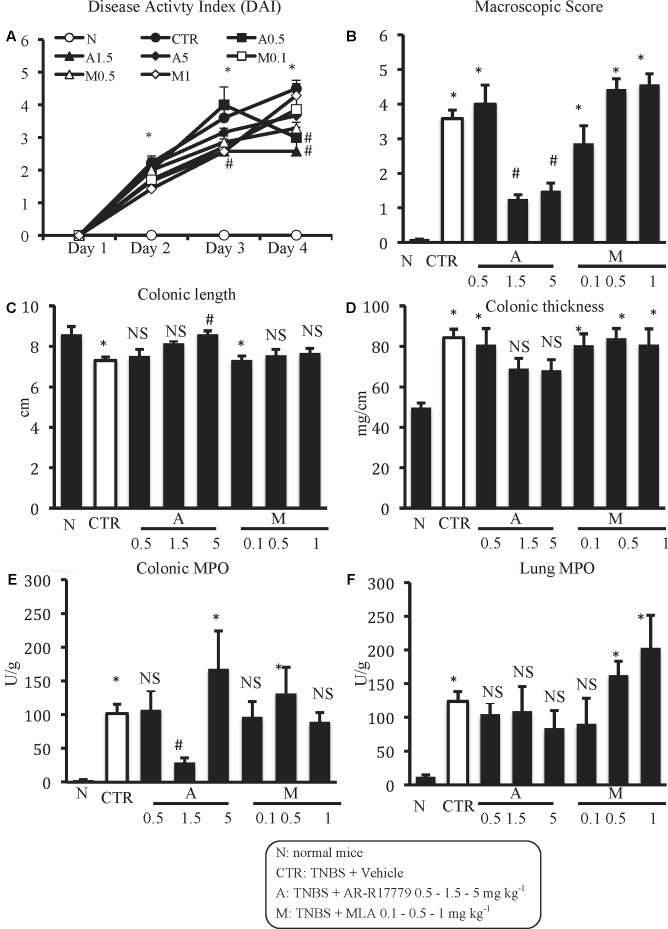
Effects of α_7_ nAChRs ligands on TNBS-induced inflammatory responses. Disease Activity Index **(A)**, macroscopic score **(B)**, colonic length **(C)**, colonic thickness **(D)**, colonic MPO **(E),** and lung MPO **(F)** activity assessed in vehicle-treated normal mice (N) and in TNBS-treated mice administered with vehicle (CTR – white bar), AR-R17779 (A) 0.5, 1.5, and 5 mg/kg, MLA (M) 0.1, 0.5 and 1 mg/kg (*n* = 7–12 independent values per group). N and CTR values were the same already represented in **Figure 1**. ^∗^*P* < 0.05 vs. N mice; ^#^*P* < 0.05 vs. CTR mice; NS: not significant vs. N and CTR mice; one-way ANOVA followed by Bonferroni’s post-test.

Among the three different tested doses, the selective α_7_ nAChRs agonist AR-R17779 evoked the most prominent effects when administered at 1.5 mg kg^-1^. In fact, DAI had been ameliorated since day 3, reflecting an attenuation of both body weight loss and diarrhea (**Figure 3A**). As regards TNBS-induced local damage, treatment with AR-R17779 1.5 mg kg^-1^ decreased MS, counteracted colonic neutrophil infiltration (**Figures 3B,E**) and weakly mitigated mucosal destruction (**Figure 2C**; histological score: 4.0 ± 0.9 in Supplementary Figure 1A).

The local beneficial effects of AR-R17779 1.5 mg kg^-1^ were confirmed by the evaluation of inflammatory cytokines levels in the colon. TNBS exposure produced a remarkable increase of IL-1β and IL-6 levels compared with N mice, whilst moderate, not significant, increase of IFN-γ and reduction of IL-10 concentrations were observed. Treatment with α7 agonist slightly reduced colonic IL-1β and remarkably lowered IL-6 levels, but did not significantly modify either IFN-γ or IL-10 concentrations (**Table 1**).

**Table 1:** Effects of AR-R17779 on cytokines levels in TNBS-induced colitis.

Cytokines	N (pg mg^-1^ proteins)	C (pg mg^-1^ proteins)	A1.5 (pg mg^-1^ proteins)
IL-1β	21.89 ± 6.82	313.9 ± 30.88^∗^	195.3 ± 36.14^NS^
IL-6	17.44 ± 1.87	375.3 ± 33.21^∗^	129.0 ± 50.77^#^
IFNγ	8.73 ± 2.97	17.38 ± 7.12^NS^	20.35 ± 6.98^NS^
IL-10	19.75 ± 3.59	15.6 ± 1.81^NS^	16.0 ± 0.91^NS^


*Colonic concentrations of IL-1β, IL-6, IFNγ, and IL-10, expressed as pg mg^*-1*^ proteins, in normal mice (N) and in TNBS-treated mice administered with vehicle (C) or AR-R17779 1.5 mg/kg (A1.5) (*n* = 7 independent values per group). ^∗^*P* < 0.05 vs. N mice; ^#^*P* < 0.05 vs. CTR mice; ^NS^: not significant vs. N and CTR mice; one-way ANOVA + Bonferroni’s post-test.*

AR-R17779 appeared ineffective when tested at 0.5 mg kg^-1^ and exhibited only sporadic protective effects at 5 mg kg^-1^. Indeed, treatment with AR-R17779 5 mg kg^-1^ attenuated MS and colonic shortening and thickening (**Figures 3B–D**) while no remarkable effects were produced on the other inflammatory parameters with respect to CTR mice.

At contrast with exogenous α7 nAChRs stimulation, treatment with α7 antagonist MLA did not substantially affect the inflammatory parameters taken into consideration, except for MS and pulmonary MPO, which deteriorated, albeit not significantly, in mice administered with MLA 0.5 and 1 mg kg^-1^ with respect to vehicle-treated mice (**Figures 3B,F**).

### AR-R17779 Attenuated TNBS-Induced Changes on Splenocytes Subpopulations

**FIGURE 4:**
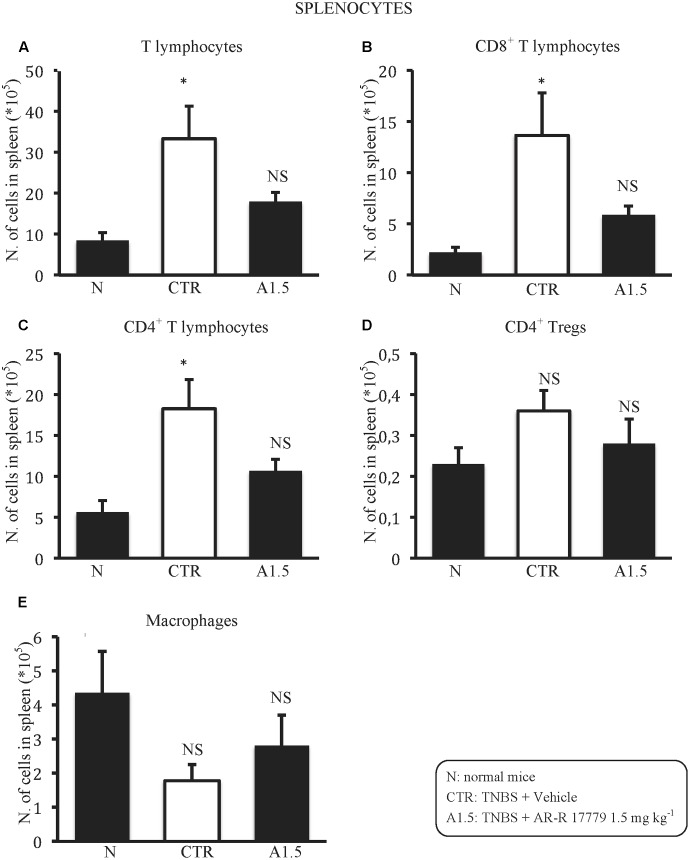
AR-R17779 attenuated TNBS-induced changes in splenocytes subpopulations. Number of T lymphocytes **(A)**, CD8^+^ **(B)**, CD4^+^ **(C)**, CD4^+^ Tregs **(D)** lymphocytes and macrophages **(E)** in the spleen excised from vehicle-treated normal mice (N) and TNBS-treated mice administered with vehicle (CTR – white bar) or AR-R17779 1.5 mg/kg (A1.5) (*n* = 7–10 independent values per group). ^∗^*P* < 0.05 vs. N mice; NS: not significant vs. N and CTR mice; one-way ANOVA followed by Bonferroni’s post-test.

In order to investigate the mechanism of action of AR-R17779, we performed the phenotypic characterization of splenocytes and MLN lymphocytes in N, CTR and A1.5 animals. TNBS instillation remarkably augmented the number of splenic T lymphocytes, both CD8^+^ and CD4^+^ and slightly reduced macrophages count: such changes were mitigated by treatment with α7 agonist (**Figure 4**). As regards MLN, macrophages could not be identified, while a moderate reduction in the number of lymphocytes, in particular of CD4^+^ T cells, was elicited by colitis (**Table 2**). The administration of AR-R17779 did not prevent TNBS-induced MLN depletion, while further diminishing the number of MLN FoxP3^+^ Tregs (**Table 2**).

**Table 2:** Effects of AR-R17779 on TNBS-induced changes in MLN subpopulations.

	MLN (^∗^10^5^ cells)			SPX-MLN (^∗^10^5^ cells)		
		
	N	C	A1.5	SPX/N	SPX/C	SPX/A1.5
T lymphocytes	16.18 ± 5.14	8.37 ± 3.41^NS^	8.35 ± 2.83^NS^	7.24 ± 1.31	3.68 ± 1.36^NS^	2.74 ± 0.38^NS^
CD8^+^	2.49 ± 0.66	2.98 ± 1.43^NS^	1.81 ± 0.52^NS^	1.95 ± 0.53	0.68 ± 0.28^NS^	0.83 ± 0.24^NS^
CD4^+^	13.23 ± 4.26	4.72 ± 1.73^∗^	5.95 ± 2.12^∗^	4.39 ± 0.69^$^	2.64 ± 0.95^NS^	1.67 ± 0.14^NS^
CD4^+^Treg	0.75 ± 0.18	0.43 ± 0.12^∗^	0.20 ± 0.04^∗#^	0.36 ± 0.06	0.12 ± 0.03^NS^	0.14 ± 0.01^NS^


*Number of T lymphocytes, CD8^+^, CD4^+^, CD4^+^ Tregs lymphocytes in MLN excised from normal mice (N) and TNBS-treated mice administered with vehicle (C) or AR-R17779 1.5 mg/kg (A1.5), non-operated (MLN) or subjected to splenectomy (SPX-MLN) (*n* = 7 independent values per group). ^∗^*P* < 0.05 vs. corresponding N mice; ^#^P < 0.05 vs. corresponding CTR mice; ^$^*P* < 0.05 vs. corresponding non-operated mice; ^NS^: not significant vs. N and CTR mice; two-way ANOVA + Bonferroni’s post-test.*

### Splenectomy Abolished the Protective Effects of AR-R17779

**FIGURE 5:**
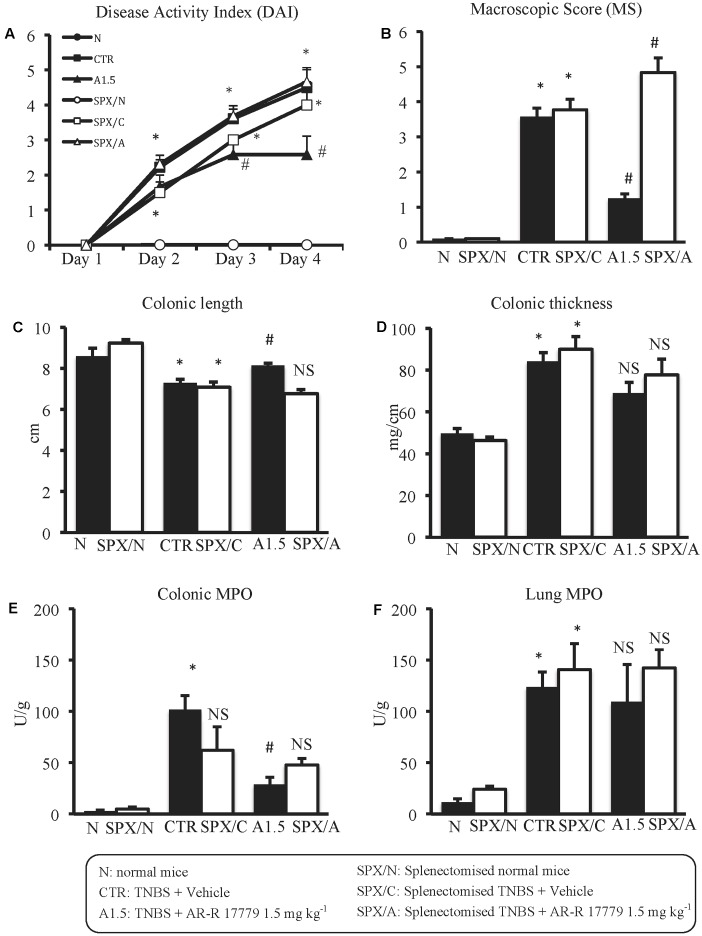
Splenectomy abolished the protective effects of AR-R17779 on TNBS-induced colitis. Disease Activity Index **(A)**, macroscopic score **(B)**, colonic length **(C)**, colonic thickness **(D)**, colonic MPO **(E)** and lung MPO **(F)** activity assessed in vehicle-treated normal mice (N; SPX/N) and in TNBS-treated mice administered with vehicle (CTR; SPX/C) or AR-R17779 1.5 mg/kg (A1.5; SPX/A), non-operated (black bars) or subjected to SPX (white bars) (*n* = 10–13 independent values per group). A1.5 values were the same already represented in **Figure 3**. ^∗^*P* < 0.05 vs. N mice; ^#^*P* < 0.05 vs. CTR mice; NS: not significant vs. N and CTR mice; two-way ANOVA followed by Bonferroni’s post-test.

In SPX mice, colitis severity was similar to that induced in non-operated animals as regards both local and systemic effects, while AR-R17779 lost its protective properties in terms of DAI and colonic granulocytic infiltration, even worsening, instead of diminishing, MS (**Figure 5**). As regards histological injury, whose score is represented in Supplementary Figure 1B, mucosal damage in vehicle-treated colitic mice (**Figure 2E**) was increased compared to SPX/N mice (**Figure 2D**) but was not prevented by AR-R17779 treatment (**Figure 2F**).

**FIGURE 6:**
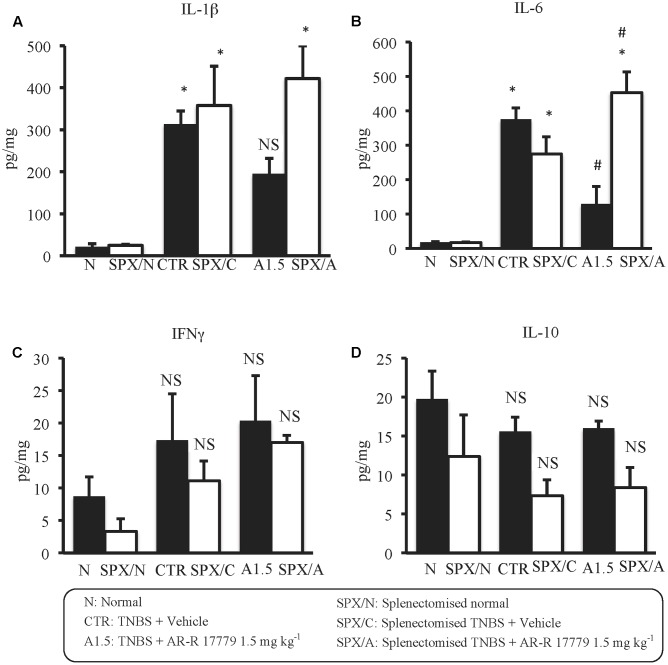
Splenectomy abolished the effects of AR-R17779 on cytokines levels in TNBS-induced colitis. Colonic concentrations of IL-1β **(A)**, IL-6 **(B)**, IFNγ **(C)**, and IL-10 **(D)** in vehicle-treated normal mice (N; SPX/N) and in TNBS-treated mice administered with vehicle (CTR; SPX/C) or AR-R17779 1.5 mg/kg (A1.5; SPX/A), non-operated (black bars) or subjected to SPX (white bars) (*n* = 7 independent values per group). ^∗^*P* < 0.05 vs. N mice; ^#^*P* < 0.05 vs. CTR mice; NS: not significant vs. N and CTR mice; two-way ANOVA followed by Bonferroni’s post-test.

Regarding cytokines levels, in SPX mice TNBS produced the same changes evoked in non-operated animals; in particular, SPX/C mice exhibited a marked rise in IL-1β and IL-6, a weak increment of IFNγ and a feeble decrease of IL-10 colonic concentrations. Interestingly, after SPX, the treatment with α_7_ agonist lost completely its protective effects against colitis, being unable to lower IL-1β concentrations and exacerbating TNBS-induced increase of IL-6 levels, while leaving unmodified IFNγ and IL-10 colonic content with respect to SPX/C mice (**Figure 6**).

With respect to MLN T cells subpopulations, SPX slightly decreased T cells number in all the experimental groups, a reduction particularly evident for CD4^+^ lymphocytes. Administration of α_7_ agonist was unable to counteract the further reduction in MLN T cells count induced by colitis (**Table 2**).

## Discussion

The results collected in the present study indicate that the exogenous stimulation of α_7_ nAChRs by AR-R17779 exerted a beneficial effect in mice subjected to TNBS-induced colitis: this protection is spleen-dependent and it apparently involves the regulation of splenic T lymphocytes responses.

Intrarectal instillation of TNBS after skin sensitization produced in mice severe colitis, characterized by progressive deterioration of their clinical conditions with consistent weight loss and diarrhea. Colonic mucosal damage, already evident at the macroscopic observation and confirmed by histological examination, was accompanied by shortening and thickening of the intestinal wall and by the increase in IL-1β and IL-6 levels; a strong neutrophil infiltration was moreover revealed both locally and remotely in the lungs. This intense inflammatory response was mirrored by the remarkable rise in the number of T lymphocytes in the spleen and by their reduction in MLN, changes supporting the strong involvement of adaptive immune responses in our model of experimental colitis. As expected, the anti-inflammatory agent sulfasalazine, conventional therapeutic agent in human IBD (Talley et al., 2011), here employed as positive control, was able to efficiently counteract most of the TNBS-induced alterations, confirming the predictive power of the adopted experimental model.

As regards the effects produced by ligands of α_4_β_2_ nAChRs, the interpretation of the data obtained is not immediately straightforward. Indeed, both the agonist TC-2403 and the antagonist dihydro-β erythroidine generally evoked feeble effects and neither of them was able to remarkably modify the severity of TNBS-induced colitis. The only parameter significantly improved was represented by animals’ clinical conditions, surprisingly ameliorated by both ligands. If, in the case of the agonist, its antinociceptive properties may come into play (Rowley et al., 2008), the beneficial action of DBE is less comprehensible: to this end, we should recall the ability of ligands, either agonists or antagonists, to desensitize and/or up-regulate nAChRs following repeated administration (Hurst et al., 2013), leading possibly to overlapping actions. As a result, no firm conclusions can be drawn on the role played by α_4_β_2_ nAChRs in our model of intestinal inflammation: it is likely that the involvement of innate immune cells like macrophages, sensitive to α_4_β_2_ nAChRs modulation (van der Zanden et al., 2009), is overridden by that of adaptive immune components, whose response to α_4_β_2_ nAChRs ligands is yet to be clarified.

With respect to the effects produced by α_7_ nAChRs ligands, we could notice that repeated administration of α_7_ antagonist MLA did not significantly modify the local and systemic inflammatory responses evoked in the model we adopted of TNBS-induced colitis. Our findings seem therefore to indicate the absence of an endogenous α_7_ nAChRs-mediated counter-inflammatory cholinergic tone, similarly to what documented by Di Giovangiulio et al. (2016), who demonstrated that the severity of DSS-induced and T-cell transfer colitis was not increased in α_7_ nAChR^-/-^ mice.

Contrarily to MLA, α_7_ nAChRs full agonist AR-R17779 was as effective as sulfasalazine in improving mice clinical conditions, in decreasing colonic macroscopic damage and pro-inflammatory cytokines levels and in preventing local neutrophil infiltration, demonstrating the remarkable protection afforded by exogenous stimulation of this receptor subtype against TNBS-induced colitis. Our results represent therefore a further clue in support of the anti-inflammatory action of α_7_ nAChRs activation and of the central role played by this receptor subtype in the CAP. Interestingly, AR-R17779 displayed a bell-shaped dose-response curve, exhibiting the greatest efficacy at the intermediate dose of 1.5 mg/kg s.c., thus confirming the tendency of α_7_ nAChRs to undergo desensitization in a dose-dependent way (Hurst et al., 2013). The findings collected are in line with those presented by Salaga et al. (2016), who demonstrated the protective effects of α_7_ nAChR partial agonist encenicline against TNBS-induced colitis, supposedly through the modulation of immune cells responses. As regards the investigation by Snoek et al. (2010) apparently denoting a lack of effect of GSK1345038A in the same experimental model, the comparison with our data could be misleading and not relevant, given the different conditions adopted (higher dose and double exposure to TNBS in our model) and the different inflammatory parameters examined by the two studies.

The beneficial effects of AR-R17779 were accompanied by its ability to attenuate the changes caused by TNBS exposure on splenic T lymphocytes, while colitis-induced MLN changes were not antagonized: this observation prompted us to speculate that the protection by the α_7_ nAChR agonist might be mediated by its immune suppressant effects on splenic T lymphocytes and by its efficacy in preventing their expansion and proliferation. Indeed, the vagal nicotinic down-regulation of splenic CD4^+^ T cells functions (Karimi et al., 2010) and the proliferative effects of α_7_ nAChRs antagonists on T cells (De Rosa et al., 2009) would point toward this mechanism of action.

Following SPX, colitis severity was not modified: in operated mice, the local and systemic inflammatory indices induced by haptenization were comparable to those of non-operated animals. Intriguingly, removal of the spleen provoked a proportional decrease in the count of MLN T lymphocytes in normal and colitic mice with respect to non-operated animals, suggesting that MLN T lymphocytes derive partly from spleen trafficking. This observation confirmed us also about the fact that changes in the number of MLN T lymphocytes do not reflect changes in the severity of colonic inflammatory responses. Notably, AR-R17779 lost completely its beneficial effects in colitic splenectomized mice, MS being even worsened by α_7_ nAChRs agonist. The spleen-dependence of the protective effect of nicotine has been already demonstrated in a model of lethal polymicrobial sepsis, where the activation of the innate immune system was considered pivotal to the development of the inflammatory process (Huston et al., 2006), but the involvement of the spleen in the modulation of adaptive immune responses by selective exogenous stimulation of α_7_ nAChRs is a novel finding.

Our results show now, for the first time, that AR-R17779 exerts beneficial effects in a model of intestinal inflammation characterized by activation of the adaptive immune system and that the spleen appears essential to the cholinergic protection. Starting from these considerations, intriguing results could arise also from the investigation of AR-R17779 effects in another model of hapten-induced colitis, such as oxazolone colitis, where the protective effect of nicotine has already been demonstrated (Galitovskiy et al., 2011) and where natural killer T cells dictate the Th2-associated cytokine pattern with respect to the Th1 profile typical of TNBS-induced colitis (Kiesler et al., 2015). On the whole, these studies will help to clarify the relationship between α_7_ nAChRs-mediated protection and splenic T cells functions and its possible implications in view of a therapeutic strategy against chronic inflammatory disorders based on the utilization of selective α_7_ nAChRs agonists.

## Author Contributions

AG, IZ, LF, and VV performed the experiments; AC carried out the histological analysis; VB analyzed the data; EB, SB, and AG designed the research, analyzed the data and wrote the paper; all the authors critically revised the manuscript, finally approved it and agreed to be accountable for all aspects of the work.

## Conflict of Interest Statement

The authors declare that the research was conducted in the absence of any commercial or financial relationships that could be construed as a potential conflict of interest.

## Abbreviations

CAP: cholinergic anti-inflammatory pathway
CTR: control
DAI: Disease Activity Index
DBE: dihydro-β-erythroidine
FCS: fetal calf serum
MLA: methyllycaconitine
MLN: mesenteric lymph nodes
MPO: myeloperoxidase
MS: macroscopic score
N: normal
nAChR: nicotinic Ach receptor
RCF: relative centrifugal force
SPX: splenectomy
TNBS: 2,4,6-trinitrobenzene sulfonic acid

## References

[B1] Bencherif M. Lovette M. Fowler K. Arrington S. Reeves L. Caldwell W. (1996). RJR-2403: a nicotinic agonist with CNS selectivity I. In vitro characterization. *J. Pharmacol. Exp. Ther.* 279 1422–1429. 8968366

[B2] Bischoff S. C. Mailer R. Pabst O. Weier G. Sedlik W. Li Z. (2009). Role of serotonin in intestinal inflammation: knock out of serotonin reuptake transporter exacerbates 2,4,6-trinitrobenzene sulfonic acid colitis in mice. *Am. J. Physiol. Gastrointest. Liver Physiol.* 296 G685–G695. 10.1152/ajpgi.90685.2008 19095763

[B3] Bonaz B. Picq C. Sinniger V. Mayol J. F. Clarençon D. (2013). Vagus nerve stimulation: from epilepsy to the cholinergic anti-inflammatory pathway. *Neurogastroenterol. Motil.* 25 208–221. 10.1111/nmo.12076 23360102

[B4] Cooper H. S. Murthy S. N. Shah R. S. Sedergran D. J. (1993). Clinico-pathological study of dextran sulfate sodium experimental murine colitis. *Lab. Invest.* 69 238–249. 8350599

[B5] Costa R. Motta E. M. Manjavachi M. N. Cola M. Calixto J. B. (2012). Activation of the alpha-7 nicotinic acetylcholine receptor (α7 nAchR) reverses referred mechanical hyperalgesia induced by colonic inflammation in mice. *Neuropharmacology* 63 798–805. 10.1016/j.neuropharm.2012.06.004 22722030

[B6] Costantini T. W. Krzyzaniak M. Cheadle G. A. Putnam J. G. Hageny A. M. Lopez N. (2012). Targeting a7 nicotinic acetylcholine receptor in the enteric nervous system: a cholinergic agonist prevents gut barrier failure after severe burn injury. *Am. J. Pathol.* 181 478–486. 10.1016/j.ajpath.2012.04.005 22688057

[B7] Curtis M. J. Bond R. A. Spina D. Ahluwalia A. Alexander S. P. A. Giembycz M. A. (2015). Experimental design and analysis and their reporting: new guidance for publication in BJP. *Br. J. Pharmacol.* 172 3461–3471. 10.1111/bph.12856 26114403 PMC4507152

[B8] De Biasi M. Dani J. A. (2011). Reward, addiction, withdrawal to nicotine. *Annu. Rev. Sci.* 34 105–130. 10.1146/annurev-neuro-061010-113734 21438686 PMC3137256

[B9] De Jonge W. J. Van Der Zanden E. P. The F. O. Bijlsma M. F. Van Westerloo D. J. Bennink R. J. (2005). Stimulation of the vagus nerve attenuates macrophage activation by activating the Jak2/STAT3 signaling pathway. *Nat. Immunol.* 6 844–851. 10.1038/ni1229 16025117

[B10] De Rosa M. J. Dionisio L. Agriello E. Bouzat C. Esandi M. C. (2009). Alpha 7 nicotinic acetylcholine receptor modulates lymphocyte activation. *Life Sci.* 85 444–449. 10.1016/j.lfs.2009.07.010 19632243

[B11] Di Giovangiulio M. Bosman G. Meroni E. Stakenborg N. Florens M. Farro G. (2016). Vagotomy affects the development of oral tolerance and increases susceptibility to develop colitis independently of α7 nicotinic receptor. *Mol. Med.* 22 464–476. 10.2119/molmed.2016.00062 27341335 PMC5072409

[B12] Downs A. M. Bond C. E. Hoover D. B. (2014). Localization of α7 nicotinic acetylcholine receptor mRNA, and protein within the cholinergic anti-inflammatory pathway. *Neuroscience* 266 178–185. 10.1016/j.neuroscience.2014.02.011 24561218

[B13] Galitovskiy V. Qian J. Chernyavsky A. I. Marchenko S. Gindi V. Edwards R. A. (2011). Cytokine-induced alterations of α7 nicotinic receptor in colonic CD4 T cells mediate dichotomous response to nicotine in murine models of Th1/Th17- versus Th2-mediated colitis. *J. Immunol.* 187 2677–2687. 10.4049/jimmunol.1002711 21784975 PMC4260719

[B14] Ge J. Tian J. Yang H. Hou L. Wang Z. He Z. (2016). Alpha7 nicotine acetylcholine receptor agonist PNU-282987 attenuates acute lung injury in a cardiopulmonary bypass model in rats. *Shock* 47 474–479. 10.1097/SHK.0000000000000744 27661000

[B15] Goverse G. Stakenborg M. Matteoli G. (2016). The intestinal cholinergic anti-inflammatory pathway. *J. Physiol.* 594 5771–5780. 10.1113/JP271537 26959627 PMC5063935

[B16] He Y. Ye Z. Q. Li X. Zhu G. S. Liu Y. Yao W. F. (2016). Alpha7 nicotinic acetylcholine receptor activation attenuated intestine-derived acute lung injury. *J. Surg. Res.* 201 258–265. 10.1016/j.jss.2015.10.046 27020805

[B17] Hijioka M. Matsushita H. Ishibashi H. Hisatsune A. Isohama Y. Katsuki H. (2012). α7 nicotinic acetylcholine receptor agonist attenuates neuropathological changes associated with intracerebral hemorrhage in mice. *Neuroscience* 222 10–19. 10.1016/j.neuroscience.2012.07.024 22820264

[B18] Holmes K. Lantz L. M. Fowlkes B. J. Schmid I. Giorgi J. V. (2001). Preparation of cells and reagents for flow cytometry. *Curr. Protoc. Immunol.* 44 5.3.1–5.3.24. 10.1002/0471142735.im0503s44 18432799

[B19] Hosur V. Loring R. H. (2011). α4β2 nicotinic receptors partially mediate anti-inflammatory effects through Janus kinase 2-signal transducer and activator of transcription 3 but not calcium or cAMP signaling. *Mol. Pharmacol.* 79 167–174. 10.1124/mol.110.066381 20943775

[B20] Hurst R. Rollema H. Bertrand D. (2013). Nicotinic acetylcholine receptors: from basic science to therapeutics. *Pharmacol. Ther.* 137 22–54. 10.1016/j.pharmthera.2012.08.012 22925690

[B21] Huston J. M. Ochani M. Rosas-Ballina M. Liao H. Ochani K. Pavlov V. A. (2006). Splenectomy inactivates the cholinergic antiinflammatory pathway during lethal endotoxemia and polymicrobial sepsis. *J. Exp. Med.* 203 1623–1628. 10.1084/jem.20052362 16785311 PMC2118357

[B22] Joh E. H. Kim D. H. (2011). Kalopanaxsaponin A ameliorates experimental colitis in mice by inhibiting IRAK-1 activation in the NF-KB and MAPK pathways. *Br. J. Pharmacol.* 162 1731–1742. 10.1111/j.1476-5381.2010.01195.x 21198552 PMC3081117

[B23] Karimi K. Bienenstock J. Wang L. Forsythe P. (2010). The vagus nerve modulates CD4+ T cell activity. *Brain Behav. Immun.* 24 316–323. 10.1016/j.bbi.2009.10.016 19887104

[B24] Kashiwagi S. Khan M. A. Yasuhara S. Goto T. Kem W. R. Tompkins R. G. (2017). Prevention of burn-induced inflammatory responses and muscle wasting by GTS-21, a specific agonist for α7 nicotinic acetylcholine receptors. *Shock* 47 61–69. 10.1097/SHK.0000000000000729 27529131 PMC5167674

[B25] Kawashima K. Fujii T. Moriwaki Y. Misawa H. Horiguchi K. (2012). Reconciling neuronally and nonneuronally derived acetylcholine in the regulation of immune function. *Ann. N. Y. Acad. Sci.* 1261 7–17. 10.1111/j.1749-6632.2012.06516.x 22823388

[B26] Kiesler P. Fuss I. J. Strober W. (2015). Experimental models of inflammatory bowel disease. *Cell. Mol. Gastroenterol. Hepatol.* 1 154–170. 10.1016/j.jcmgh.2015.01.006 26000334 PMC4435576

[B27] Kilkenny C. Browne W. J. Cuthill I. C. Emerson M. Altman D. G. (2010). Improving bioscience research reporting: the ARRIVE guidelines for reporting animal research. *PLOS Biol.* 8:e1000412. 10.1371/journal.pbio.1000412 20613859 PMC2893951

[B28] Krawisz J. E. Sharon P. Stenson W. F. (1984). Quantitative assay for acute intestinal inflammation based on myeloperoxidase activity. Assessment of inflammation in rat and hamster models. *Gastroenterology* 87 1344–1350. 6092199

[B29] Kruisbeek A. M. (2001). Isolation of mouse mononuclear cells. *Curr. Protoc. Immunol.* 39 3.1.1–3.1.5. 10.1002/0471142735.im0301s39 18432779

[B30] Li F. Chen Z. Pan Q. Fu S. Lin F. Ren H. (2013). The protective effect of PNU-282987, a selective a7 nicotinic acetylcholine receptor agonist, on the hepatic ischemia-reperfusion injury is associated with the inhibition of high-mobility group box 1 protein expression and nuclear factor kB activation in mice. *Shock* 39 197–203. 23324890 10.1097/SHK.0b013e31827aa1f6

[B31] Lopez M. C. (2007). Fluorescence microscopy and flow cytometric analysis of Peyer’s patches and intestinal immune cells. *Curr. Protoc. Immunol.* 33 18.13.1–18.13.20. 10.1002/0471140856.tx1813s33 23045140

[B32] Mahida Y. R. Wu K. Jewell D. P. (1989). Enhanced production of interleukin 1-beta by mononuclear cells isolated from mucosa with active ulcerative colitis of Crohn’s disease. *Gut* 30 835–838. 10.1136/gut.30.6.835 2787769 PMC1434123

[B33] Martelli D. McKinley M. J. McAllen R. M. (2014). The cholinergic anti-inflammatory pathway: a critical review. *Auton. Neurosci.* 182 65–69. 10.1016/j.autneu.2013.12.007 24411268

[B34] Matsunaga K. Klein T. W. Friedman H. Yamamoto Y. (2001). Involvement of nicotinic acetylcholine receptors in suppression of antimicrobial activity and cytokine responses of alveolar macrophages to *Legionella pneumophila* infection by nicotine. *J. Immunol.* 167 6518–6524. 10.4049/jimmunol.167.11.6518 11714820

[B35] Moore-Olufemi S. D. Kozar R. A. Moore F. A. Sato N. Hassoun H. T. Cox C. S. Jr. (2005). Ischemic preconditioning protects against gut dysfunction and mucosal injury after ischemia/reperfusion injury. *Shock* 23 258–263. 15718925

[B36] Mullen G. Napier J. Balestra M. DeCory T. Hale G. Macor J. (2000). (-)-Spiro[1-azabicyclo[2.2.2] octane-3,5’-oxazolidin-2’-one], a conformationally restricted analogue of acetylcholine, is a highly selective full agonist at the alpha 7 nicotinic acetylcholine receptor. *J. Med. Chem.* 43 4045–4050. 10.1021/jm000249r 11063601

[B37] Niimi K. Nishioka C. Miyamoto T. Takahashi E. Miyoshi I. Itakura C. (2013). Improvement of spontaneous alternation behavior deficit by activation of α4β2 nicotinic acetylcholine receptor signaling in the ganglioside GM3-deficient mice. *Biomed. Res.* 34 189–195. 10.2220/biomedres.34.189 23995055

[B38] Nullens S. Staessens M. Peleman C. Schrijvers D. M. Malhotra-Kumar S. Francque S. (2016). Effect of GTS-21, an alpha7 nicotinic acetylcholine receptor agonist, on CLP-induced inflammatory, gastrointestinal motility, and colonic permeability changes in mice. *Shock* 45 450–459. 10.1097/SHK.0000000000000519 26618987

[B39] Olofsson P. S. Rosas-Ballina M. Levine Y. A. Tracey K. J. (2012). Rethinking inflammation: neural circuits in the regulation of immunity. *Immunol. Rev.* 248 188–204. 10.1111/j.1600-065X.2012.01138.x 22725962 PMC4536565

[B40] Pinheiro N. M. Santana F. P. Almeida R. R. Guerreiro M. Martins M. A. Caperuto L. C. (2017). Acute lung injury is reduced by the α7 nAChR agonist PNU-282987 through changes in the macrophage profile. *FASEB J.* 31 320–332. 10.1096/fj.201600431R 27729414

[B41] Rapalli A. Bertoni S. Arcaro V. Saccani F. Grandi A. Vivo V. (2016). Dual role of endogenous serotonin in 2,4,6-Trinitrobenzene Sulfonic acid-induced colitis. *Front. Pharmacol.* 7:68. 10.3389/fphar.2016.00068 27047383 PMC4802166

[B42] Rowley T. J. Payappilly J. Lu J. Flood P. (2008). The antinociceptive response to nicotinic agonists in a mouse model of postoperative pain. *Anesth. Analg.* 107 1052–1057. 10.1213/ane.0b013e318165e0c0 18713928

[B43] Saeed R. W. Varma S. Peng-Nemeroff T. Sherry B. Balakhaneh D. Huston J. (2005). Cholinergic stimulation blocks endothelial cell activation and leukocyte recruitment during inflammation. *J. Exp. Med.* 201 1113–1123. 10.1084/jem.20040463 15809354 PMC2213139

[B44] Salaga M. Blomster L. V. Piechota-Polanczyk M. Zielinska M. Jacenik D. Cygankiewicz A. I. (2016). Encenicline, an α7 nicotinic acetylcholine receptor partial agonist, reduces immune cell infiltration in the colon and improves experimental colitis in mice. *J. Pharmacol. Exp. Ther.* 356 157–169. 10.1124/jpet.115.228205 26462538

[B45] Snoek S. A. Verstege M. I. Van der Zanden E. P. Deeks N. Bulmer D. C. Skynner M. (2010). Selective α7 nicotinic acetylcholine receptor agonists worsen disease in experimental colitis. *Br. J. Pharmacol.* 160 322–333. 10.1111/j.1476-5381.2010.00699.x 20423343 PMC2874854

[B46] Talley N. J. Abreu M. T. Achkar J. P. Bernstein C. N. Dubinsky M. C. Hanauer S. B. (2011). An evidence-based systematic review on medical therapies for inflammatory bowel disease. *Am. J. Gastroenterol.* 106 S2–S25. 10.1038/ajg.2011.58 21472012

[B47] Te Velde A. A. Verstege M. I. Hommes D. W. (2006). Critical appraisal of the current practice in murine TNBS-induced colitis. *Inflamm. Bowel Dis.* 12 995–999. 10.1097/01.mib.0000227817.54969.5e 17012970

[B48] The F. O. Boeckxstaens G. E. Snoek S. A. Cash J. A. Bennink R. Larosa G. J. (2007). Activation of the cholinergic anti-inflammatory pathway ameliorates postoperative ileus in mice. *Gastroenterology* 133 1219–1228. 10.1053/j.gastro.2007.07.022 17919496

[B49] van der Zanden E. P. Snoek S. A. Heinsbroek S. E. Stanisor O. I. Verseijden C. Boeckxstaens G. E. (2009). Vagus nerve activity augments intestinal macrophage phagocytosis via nicotinic acetylcholine receptor alpha4beta2. *Gastroenterology* 13 1029–1039. 10.1053/j.gastro.2009.04.057 19427310

[B50] Waldner M. J. Neurath M. F. (2009). Chemically induced mouse models of colitis. *Curr. Protoc. Pharmacol.* 46 5.55.1–5.55.15. 10.1002/0471141755.ph0555s46 22294401

[B51] Wang H. Yu M. Ochani M. Amella C. A. Tanovic M. Susarla S. (2003). Nicotinic acetylcholine receptor α7 subunit is an essential regulator of inflammation. *Nature* 421 384–388. 10.1038/nature01339 12508119

[B52] Ward J. Cockcroft V. Lunt G. Smillie F. S. Wonnacott S. (1990). Methyllycaconitine: a selective probe for neuronal α-bungarotoxin binding sites. *FEBS Lett.* 270 45–48. 10.1016/0014-5793(90)81231-C 2226787

